# Lymphatic mapping and sentinel node biopsy in ovarian tumors: a study using intra-operative Tc-99m-Phytate and lymphoscintigraphy imaging

**DOI:** 10.1186/s13048-016-0265-4

**Published:** 2016-09-07

**Authors:** Malihe Hassanzadeh, Elham Hosseini Farahabadi, Zohreh Yousefi, Sima Kadkhodayan, Leili Zarifmahmoudi, Ramin Sadeghi

**Affiliations:** 1 Women’s Health Research Center, Mashhad University of Medical Sciences, Mashhad, Iran; 2 Nuclear Medicine Research Center, Mashhad University of Medical Sciences, Mashhad, Iran

**Keywords:** Ovary, Ovarian tumor, Sentinel, Lymphoscintigraphy, Lymphatic mapping, Blue dye

## Abstract

**Background:**

Experience on sentinel node mapping in ovarian tumors is very limited. We evaluated the sentinel node concept in ovarian tumors using intra-operativeTc-99m-Phytate injection and lymphoscintigraphy imaging.

**Methods:**

Thirty-five patients with a pelvic mass due to an ovarian pathology were included in the study. The radiotracer was injected just after laparotomy and before removal of the tumor either beneath the normal cortex (10 patients) or in the utero-ovarian and suspensory ligaments of the ovary just beneath the peritoneum two injections of the radiotracer (25 patients). For malignant masses, the sentinel nodes were identified using a hand held gamma probe. Then standard pelvic and para-aortic lymphadenectomy was performed. In case of benign pathologies or borderline ovarian tumors on frozen section, lymphadenectomy was not performed. The morning after surgery, all patients were sent for lymphoscintigraphy imaging of the abdomen and pelvis.

**Results:**

Sentinel node was identified only in 4 patients of the cortical injection group. At least one sentinel node could be identified in 21 patients of the sub-peritoneal group. Sentinel nodes were identified only in the para-aortic area in 21, pelvic/para-aortic areas in 2, and pelvic only area in 2 patients. Three patients had lymph node involvement and all had involved sentinel nodes (no false negative case).

**Conclusion:**

Sentinel node mapping using intra-operative injection of the radiotracer (in the utero-ovarian and suspensory ligaments of the ovary just beneath the peritoneum) is feasible in ovarian tumors. Technical aspects of this method should be explored in larger multicenter studies in the future.

## Background

Epithelial ovarian cancer is the most common ovarian malignancy and is the leading cause of death from gynecological cancers in the United States [1, 2]. About a third of epithelial ovarian cancers presents in an early stage [3]. Para-aortic and pelvic lymph node dissection is the recommended procedure for lymph node staging of epithelial ovarian cancers [4]. However routine lymph node dissection did not show any survival benefit even in advanced ovarian cancers [5]. Incidence of lymph node involvement in the early ovarian cancers (stage I) is also low (about 10–20 %) [6] and majority of patients with early ovarian cancer would not benefit from routine lymphadenectomy while being subject to its unwanted consequences such as prolonged hospitalization, more blood loss, and longer surgical time [7, 8].

In the last two decades, sentinel node mapping has been introduced to the surgical management of many solid tumors. Although lymphatic mapping has been applied successfully for gynecological and urological cancers [9–14], it has not been adequately evaluated for ovarian cancers in the medical literature: To the extent of our knowledge only four studies thus far is available in this regard [15–18].

In the current study, we evaluated the sentinel node concept in ovarian tumors using intra-operativeTc-99m-Phytate injection and lymphoscintigraphy imaging.

## Methods

During a period from Jan 2010 to Oct 2014, 35 patients with a pelvic mass diagnosed to be due to an ovarian pathology were eligible to be included in the study. All patients provided written informed consent before enrollment in the study, and the study was approved by the Local Ethical Committee of Mashhad University of Medical Sciences under the approval number of 931331. Pregnant or lactating patients, patients with the history of previous surgery on one or both ovaries; previous lymph node surgery in the pelvic or para-aortic areas; a history of any malignant tumor in the abdominal or pelvic cavities were excluded from the study.

### Sentinel node mapping

**Fig. 1:**
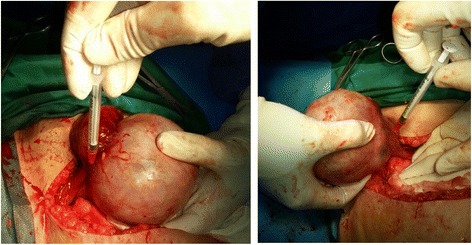
Radiotracer injection in the utero-ovarian and suspensory ligaments of the ovary just beneath the peritoneum

We used Tc-99m-Phytate for lymphatic mapping in all patients. The radiotracer was injected just after laparotomy and before removal of the tumoral ovary. The first 10 patients received two injections of the radiotracer (1 mCi/0.2 mL/injection) beneath the normal cortex of the ovary. The remainder of the patients received two injections of the radiotracer (1 mCi/0.2 mL each) in the utero-ovarian and suspensory ligaments of the ovary just beneath the peritoneum close to the diseased ovary (Fig. 1). In four patients of the second group, patent blue V dye was also injected in the same location as the radiotracer (0.2 cc/injection).

The surgeon waited for 10 min interval before removal of the adnexal mass in order to give the radiotracer enough time to move in the lymphatics. The adnexal mass was then sent to the pathologist for frozen section evaluation. For malignant masses, the sentinel nodes were identified using a hand held gamma probe (EUROPROBE, Lyon, France) by searching the para-aortic and pelvic areas. Any lymph node with 3 times more count than the background was considered a sentinel node and was harvested separately for histopathological evaluation using H&E staining. Then standard pelvic and para-aortic lymphadenectomy was performed.

In case of benign pathologies or borderline ovarian tumors on frozen section, lymphadenectomy was not performed. However the gamma probe was used to locate any hot area in the pelvic and par-aortic areas and the locations of any hot spots were recorded.

The morning after surgery, all patients were sent to the nuclear medicine ward for lymphoscintigraphy imaging of the abdomen and pelvis. Anterior and lateral views (5 min/image) of the abdomen and pelvis were taken by a dual head variable angle gamma camera (E.CAM, SIEMENS, Germany) equipped with low energy all-purpose collimator and Tc-99m photopeak [19]. The images were interpreted by two nuclear medicine specialists familiar with sentinel node mapping and the results were correlated with the intra-operative findings.

## Results

Overall 35 patients entered the study. Table 1 shows the characteristics of the patients. Ten patients had cortical injection of the tracer (3 with malignant and 7 with benign ovarian pathology). Sentinel node was identified only in 4 patients of this group which amounts to 40 % detection rate (2 with benign and 2 with malignant pathology). Twenty-five patients received sub-peritoneal ligament injection of the mapping material (10 with malignant, one with borderline pathology and 13 with benign pathology). At least one sentinel node could be identified in 21 patient of this group which amounts to 84 % detection rate (11 with malignant and 10 with benign pathology).

**Table 1:** Characteristics of the included patients

N	Age/tumor side/tumor size (cm)	Injection location	Final pathological results	Number and location of sentinel nodes on lymphoscintigraphy findings	Comments
1	28/left/12 × 8	L	Benign follicular cyst	Two/para-aortic	–
2	16/right/7 × 7	C	Struma ovarii	Three/para-aortic	–
3	54/left/20 × 25	L	Serous cysadenofibroma	One/para-aortic	–
4	35/left/10 × 9	L	Mature teratoma	Two/para-aortic	–
5	45/left/20 × 15	C	Granulosa cell tumor	No remaining activity in the pelvis and abdomen	TAH + BSO + APAL: Two hot sentinel nodes were identified during surgery in the para-aortic area. Sentinel nodes were not involved. No other lymph node involvement.
6	42/right/15 × 10	L	Mature teratoma with ovarian torsion	None	–
7	51/left/10 × 10	L	Benign papillary serous cyst with ovarian torsion	None	–
8	17/left/7 × 7	L	Benign serous cyst	Two/para-aortic	–
9	26/left/10 × 5	L	Benign luteal cyst	One/para-aortic	–
10	32/right/8 × 10	L	Mucinous adenocarcinoma	No remaining activity in the pelvis and abdomen	TAH + BSO + APAL: Three hot sentinel nodes were identified during surgery in the para-aortic area. None were involved. No lymph node involvement.
11	52/right/12 × 15	L	Borderline serous cystadenoma	No remaining activity in the pelvis and abdomen	TAH + BSO + APAL: One hot sentinel node was identified during surgery in the para-aortic area. None were involved. No lymph node involvement.
12	59/right/6 × 8	C	Mature teratoma	Two/para-aortic	–
13	56/right/12 × 9	C	Papillary serous adenocarcinoma	No remaining activity in the pelvis and abdomen	TAH + BSO + APAL: No hot sentinel node was identified during surgery in the para-aortic area. No lymph node involvement.
14	36/left/7 × 6	L	Mature teratoma with ovarian torsion	None	–
15	47/right/5 × 7	L	Papillary serous adenocarcinoma	No remaining activity in the pelvis and abdomen	TAH + BSO + APAL: Two hot sentinel nodes was identified during surgery in the para-aortic (one) and pelvic areas (one in the internal iliac area). None were involved. No lymph node involvement.
16	42/right/12 × 13	C	Thechoma	One/Para-aortic	–
17	31/right/6 × 6	L	Benign serous cyst	One/Para-aortic	–
18	42/right/6 × 8	C	Benign mucinous cystadenoma with ovarian torsion	None	–
19	60/Bilateral/10 × 8;6 × 8	L	Papillary serous adenocarcinoma	No remaining activity in the pelvis and abdomen	TAH + BSO + APAL: Three hot sentinel nodes was identified during surgery in the para-aortic (two) and pelvic areas (one in the obturator). Both were involved. No other lymph node involvement.
20	42/right/10 × 10	C	Benign mucinous cystadenoma	None	–
21	68/left/6 × 8	L	Benign mucinous cystadenoma	One/Para-aortic	–
22	40/left/6 × 8	L	Benign mucinous cystadenoma	One/Para-aortic	–
23	26/left/6 × 5	C	Granulosa cell tumor	No remaining activity in the pelvis and abdomen	TAH + BSO + APAL: The hot sentinel node was identified during surgery in the para-aortic area. Sentinel node was not involved. No other lymph node involvement.
24	45/left/7 × 6	L	Papillary serous adenocarcinoma	No remaining activity in the pelvis and abdomen	TAH + BSO + APAL: Two hot sentinel nodes were identified during surgery in the para-aortic (one) and external iliac (one) areas. Sentinel nodes were not involved. No other lymph node involvement.
25	22/right/10 × 11	L	Benign serous cyst	One/Para-aortic	–
26	57/right/8 × 9	L	Papillary serous adenocarcinoma	No remaining activity in the pelvis and abdomen	TAH + BSO + APAL: The hot sentinel node was identified during surgery in the para-aortic area. Sentinel node was not involved. No other lymph node involvement.
27	43/Bilateral/11 × 12;10 × 6	L	Papillary serous adenocarcinoma	No remaining activity in the pelvis and abdomen	TAH + BSO + APAL: The hot sentinel nodes were identified during surgery in the para-aortic (two sentinel nodes) and pelvic areas (right common iliac). None were involved. No other lymph node involvement.
28	36/right/11 × 10	L	Benign serous cyst	One/Para-aortic	–
29	35/Bilateral/10 × 10;10 × 7	L	Papillary serous adenocarcinoma	No remaining activity in the pelvis and abdomen	TAH + BSO + APAL: Two hot (and blue) sentinel nodes were identified during surgery in the para-aortic area. One sentinel node was pathologically involved. No other lymph node involvement.
30	50/right/7 × 8	L	Papillary serous adenocarcinoma	No remaining activity in the pelvis and abdomen	TAH + BSO + APAL: The hot (and blue) sentinel node was identified during surgery in the pelvic area (external iliac). Sentinel node was not involved. No other lymph node involvement.
31	43/Right/10 × 10	L	Papillary serous adenocarcinoma	No remaining activity in the pelvis and abdomen	TAH + BSO + APAL: The hot (and blue) sentinel node was identified during surgery in the pelvic area (external iliac). Sentinel node was not involved. No other lymph node involvement.
32	36/Right/10 × 12	L	Benign mucinous cystadenoma	One/Para-aortic	–
33	35/Bilateral/10 × 8;8 × 8	L	Papillary serous adenocarcinoma	No remaining activity in the pelvis and abdomen	TAH + BSO + APAL: Two hot (and blue) sentinel nodes were identified during surgery in the para-aortic area. One of the sentinel nodes was involved. One non-sentinel node in the para-aortic was also involved.
34	45/Left/7 × 7	C	Benign serous cyst	None	–
35	37/Left/8 × 8	C	Benign serous cyst	None	–

Four patients had ovarian torsion in addition to their underlying pathology in the ovary. No sentinel node could be identified in these patients.

Sentinel nodes were identified only in the para-aortic area in all 12 patients with benign pathology and successful lymphatic mapping. Sentinel nodes were identified in para-aortic area in 9 and pelvic/para-aortic areas in 2 patients and pelvic only area in 2 patients with malignant pathology.

**Fig. 2:**
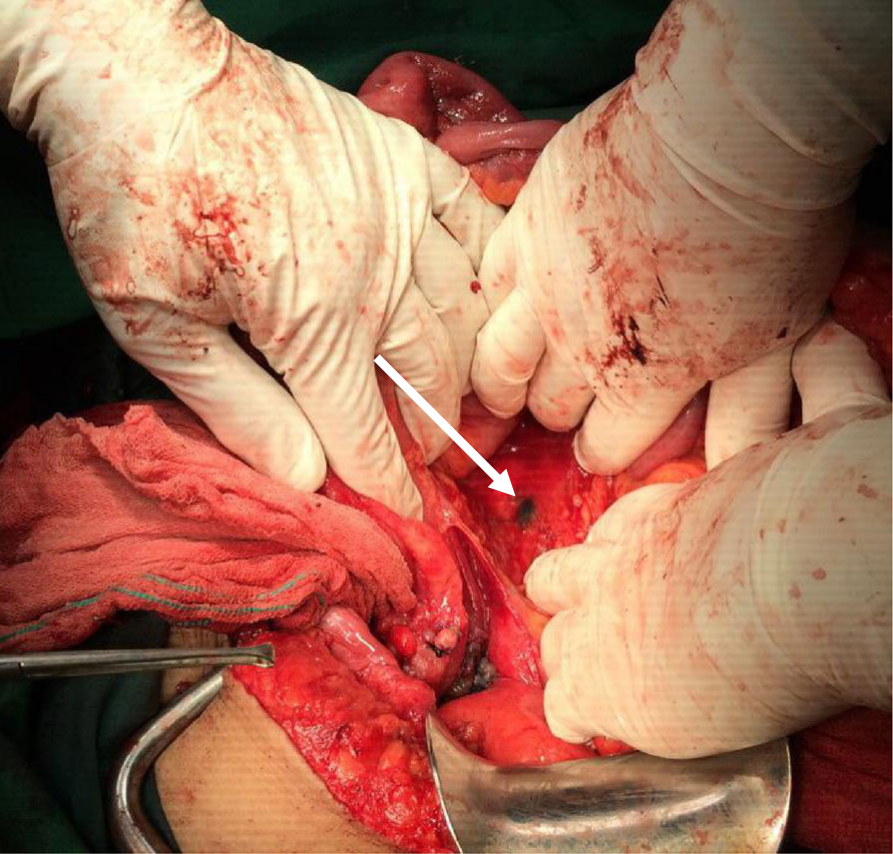
A *blue* para-aortic sentinel node could be identified in this patient intra-operatively (*arrow*)

In four patients who received blue dye injection in addition to radiotracer, all sentinel nodes were hot/blue. Figure 2 shows a blue para-aortic sentinel node in one of these patients.

Three patients with malignant pathology had lymph node involvement and all had involved sentinel nodes on pathology (100 % sensitivity and no false negative case). No adverse reaction to the radiotracer or blue dye was observed in our patients.

**Fig. 3:**
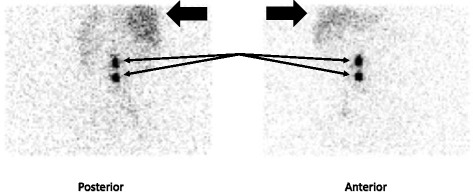
Planar anterior/posterior lymphoscintigraphy images of a patient. Two para-aortic sentinel nodes are marked by *arrows*. Activity in the liver is also apparent (*black arrows*)

Figure 3 shows lymphoscintigraphy images of patient number 8.

## Discussion

Thus far, only limited studies on lymphatic mapping in ovarian cancer have been published. Table 2 summarizes the data of these studies in addition to the current one. Two studies were done on normal ovaries of patients who underwent laparotomy for other reasons [15, 16]. Both studies reported a high sentinel node detection rate. Vanneaville et al. study used a laparoscopic mesovarian radiotracer injections in the normal ovaries of 14 patients who were treated for benign ovarian cysts or were candidate of tubal ligation. Sentinel nodes were detected 4–6 h post injection by planar lymphoscintigraphy. In 12 patients sentinel nodes could be identified (4 para-aortic and 8 pelvic/para-aortic). Isolated para-aortic drainage was more prominent in the post-menopausal (75 %) as compared to the pre-menopausal patients (12.5 %) [18, 20].

**Table 2:** Summary of the studies on lymphatic mapping of the ovaries

First author/year	Included patients	Mapping material	Injection site	Main results
Kleppe/2014	21 patients diagnosed with a pelvic mass suggestive of a malignant ovarian tumor	Blue dye/Radiotracer	On the dorsal and ventral side of the proper ovarian ligament and the suspensory ligament, close to the ovary and just underneath the peritoneum	Al least one sentinel node could be identified in all patients (100 % detection rate). Para-aortic region only in 67 %, pelvic region only in 9 %, and in both in 24 %. No false negative case
Negishi/2004	11 women with endometrial or fallopian tube tumors	Activated Charcoal	Into the unilateral cortex of the ovary	Sentinel node could be identified in all patients: para-aortic in all patients, common iliac node in three, and external iliac in one
Nyberg/2011	16 patients with high-risk uterine cancer and normal postmenopausal ovaries	Blue dye/Radiotracer	Slow injection near the hilum of one ovary	Sentinel node could be identified in 15 patients (93.75 % detection rate). All were located in the para-aortic area.
Vanneaville/1991	14 patient who were investigated by laparoscopy, either for ablation of a benign ovarian cyst or for tubal ligation	Radiotracer	Injection into the mesovarium of the normal ovaries during laparoscopy.	Lymphatic drainage could be discerned in 12 patients. Isolated para-aortic sentinel nodes in 4, combined pelvic/para-aortic sentinel nodes in 8.
			Lymphatic drainage was investigated by lymphoscintigraphy 4–6 h post-injection.	Isolated para-aortic drainage was more prominent in the post-menopausal (75 %) as compared to the pre-menopausal patients (12.5 %).
The current study	35 patients with ovarian tumors	Radiotracer	Sub-cortical in 11 and sub-peritoneal (ovarian and suspensory ligaments) in 24	Detection rate of 40 % in the sub-cortical and 84 % in the sub-peritoneal group. Sentinel nodes were identified in 21 patient in the para-aortic area only and in 4 in the pelvic/para-aortic area. No false negative case was identified

An important study published by Kleppe et al. reported the first experience on sentinel node mapping in ovarian tumors. They used radiotracer/blue dye injection into the proper ovarian ligament and the suspensory ligament and reported 100 % detection rate and sensitivity [17, 21].

Our study showed that intra-operative injection of radiotracer is a feasible method for lymphatic mapping and sentinel node biopsy in ovarian tumors. Intra-operative injection of sentinel node mapping materials has been reported to be very successful for lymphatic mapping in other solid tumors too; such as lung, gynecological, and urological tumors [11, 12, 22]. This is possible due to very rapid movement of the radiotracer in the lymphatic vessels especially those with small particle size [23–25]. In the current study we used Tc-99m Phytate which has a small particle size with rapid lymphatic movement as shown in other studies before [26–30]. Negishi et al., Nyberg et al., and Kleppe st al. used 10 min, 10–21 min, and 15 min after injection, respectively. All these three studies had excellent sentinel node detection rate. We waited for 10 min after injection with comparable detection rate too. Overall, it seem that 10 to 15 min is an optimal time to wait after mapping material injection in ovarian cancers.

Two injection methods were used in our study: sub-cortical vs. sub-peritoneal. Subcortical injection of the radiotracer was not successful enough (40 % detection rate). Finding normal cortex in the tumoral ovaries can be very hard and carries the risk of tumor puncture and it seems that sub-cortical injection of the mapping material is not a viable method for lymphatic mapping in ovarian cancers. On the other hand injection of the tracer beneath the peritoneum of the suspensory and utero-ovarian ligaments was highly successful for lymphatic mapping (84 % detection rate). All sentinel node detection failure in this group occurred in patients with ovarian torsion (3 patients) which can disrupt the lymphatic flow of the ovaries. Our results are in accordance with Kleppe et al. report as they also had a very high detection rate using the same injection method as we used in our study [17, 21].

We used a combination method (blue dye/radiotracer) in four patients and all four patients had blue para-aortic sentinel nodes. Adding blue dye to radiotracer (dual mapping method) has been shown to be an effective method for decreasing sentinel node detection failure as well as false negative cases in many tumors [31–33]. However, Kleppe et al. showed that blue dye was successful only in 2 patients out of 6 with retro-peritoneal exploration and ascribed it to the long time between blue dye injection and exploration [17]. Considering the possibility of adverse reactions to blue dyes [34, 35], the added value of blue dye injection should be evaluated in more detail in the future studies.

Imaging of the sentinel nodes using lymphoscintigraphy is an important method which is integrated in lymphatic mapping of the solid tumors [19, 30, 36–39]. We also used lymphoscintigraphy for all patients post-operatively. For patients with benign pathology; who did not underwent lymph node dissection, this is the only method to show the exact location of the sentinel nodes. For patients with malignant tumors, lymphoscintigraphy could confirm the complete resection of the sentinel nodes. Kleppe et al. didn’t use lymphoscintigraphy and for benign ovarian tumors relative intra-operative count of the para-aortic or pelvic areas was used as the surrogate of the sentinel node detection. This is a limitation of Kleppe et al. study which we avoided by adding post-operative lymphoscintigraphy to our study [17, 21]. In the future studies, application of intra-operative gamma cameras as well as SPECT/CT methods should be evaluated for sentinel node mapping in ovarian cancers.

The location of sentinel nodes in our study was also in accordance with the known primary lymphatic drainage pattern of the ovaries [18]: isolated para-aortic drainage in 84 %, isolated pelvic drainage in 8 % and combined pelvic/para-aortic in 8 % of the patients with successful lymphatic mapping. The variable lymphatic drainage pattern of the ovaries can be problematic in sentinel node mapping. Intra-operative gamma camera and pre-operative SPECT/CT can be of particular use in localization of the ovarian sentinel nodes and future studies should investigate these flourishing technologies.

False negative rate is another important index of the sentinel node mapping studies and should be considered alongside the detection rate in all sentinel node mapping feasibility study [40]. In the Kleppe et al. study, only one patient had involved nodes which were detected by sentinel node mapping [17]. In our study, three patients had involved lymph nodes and all were correctly identified by sentinel node biopsy (false negative rate of 0 % which means 100 % sensitivity). This is promising, however more studies with larger sample size is needed to confirm the results of our study. Specificity is not reported in our study as specificity of sentinel node mapping is always 100 % and no false positive case is possible. As sentinel node is a part of the lymphatic basin, pathologically involved sentinel node invariably means involved lymphatic basin and false positive case is impossible.

If the results of our study would be corroborated in the larger multicenter trials, para-aortic and pelvic lymph node dissection can be omitted in patients with non-involved sentinel nodes.

The radiation safety of sentinel node mapping has been addressed before in several studies [41]. Radiation dose to the patients is very low as systemic absorption of the tracer is minimal and the injection site is removed from the body during surgery [42]. Radiation to the surgical and nuclear medicine staff as well as the pathologists is also well below the ICRP threshold limit [43].

## Conclusion

Sentinel node mapping using intra-operative injection of the radiotracer is feasible in ovarian tumors. It seems to be an accurate method for detection of the patients with involved lymph nodes. Tracer injection in the utero-ovarian and suspensory ligaments of the ovary just beneath the peritoneum seems to be an efficient method for lymphatic mapping, however technical aspects of this method should be explored in larger multicenter studies in the future.
